# Longitudinal Changes in Plasma Caspase-1 and Caspase-3 during the First 2 Years of HIV-1 Infection in CD4_Low_ and CD4_High_ Patient Groups

**DOI:** 10.1371/journal.pone.0121011

**Published:** 2015-03-25

**Authors:** Jingjing Song, Yanmei Jiao, Tong Zhang, Yonghong Zhang, Xiaojie Huang, Hongjun Li, Hao Wu

**Affiliations:** Beijing You’an Hospital, Capital Medical University, Beijing, 100069, China; University of Colorado Denver, UNITED STATES

## Abstract

Over 95% of CD4 cell death occurs by Caspase-1-mediated pyroptosis during HIV infection. Caspase-3-mediated apoptosis accounts for the death in a small proportion of infected CD4 cells. To date, there have been no reports on the dynamics of Caspase-1 and Caspase-3 and their relationship with disease progression in acute HIV-1 infection. In this study, two distinct HIV-1 patient groups were enrolled. The CD4_High_ group maintained a CD4 level above450 cells/μl while CD4 levels in the CD4_Low_ group dropped below 250 cells/μl within 2 years after infection. Blood samples were collected at 1, 2, 3, 4, 6, 12 and 24 months after HIV infection. Plasma Caspase-1 and Caspase-3 levels in the two patients groups were determined by a single-step ELISA using commercially available monoclonal antibodies. The results showed that Caspase-1 and Caspase-3 levels in the CD4_High_ group increased rapidly and then decreased within a short time during early HIV-1 infection. In contrast, Caspase-1 and Caspase-3 levels in the CD4_Low_ group were obviously increased after 1 year of HIV-1 infection.

## Introduction

Despite extensive efforts over the past quarter-century, the mechanism by which CD4 T cells are depleted in HIV-infected hosts remains poorly understood; however, apoptosis has been proposed to be a key mechanism. Doitsh and colleagues demonstrated that more than 95% of CD4+ T cells that die following HIV-1 infection are quiescent cells that undergo pyroptosis [1]. Only a small proportion of the dying cells were activated, productively infected CD4 T cells undergoing apoptosis [1]. Apoptosis depends on the activation of the cell-signaling molecule, caspase-3, but pyroptosis is triggered by the inflammasome-activated caspase-1 [1–5]. We investigated pyroptosis and apoptosis using Caspase-1 and Caspase-3 in patients with HIV infection. To date, there have been no reports on Caspase-1 and Caspase-3 dynamics within the first 2 years after HIV-1 infection. To determine and compare the dynamics of Caspase-1 and Caspase-3 in the plasma within the first 2 years after HIV-1 infection, we measured and compared plasma caspase-1 and Caspase-3 levels in two distinct patient groups and found that caspase-1 and Caspase-3 dynamics are different between the two groups.

## Patients and Methods

### Patients

The patients in this study were from an ongoing prospective clinical study cohort of acute HIV-1-infected individuals in Beijing [6–10]. Starting in October 2006, MSM (men who have sex with men) were enrolled in a longitudinal prospective study cohort if they were at least 18 years old and HIV-negative at baseline. After enrollment, these HIV-negative men were monitored every 2 months for plasma HIV antibodies, HIV RNA levels, and clinical signs of acute infection. Whole blood specimens were collected at 1, 2, 3, 4 and 6 months and then every 3 months thereafter from the detection of seroconversion, and plasma, serum and peripheral blood mononuclear cells (PBMCs) were isolated. Twelve patients who were recently infected with HIV-1 were enrolled into our study. The patients were divided into the following two groups with significant disease progression: one group of five patients (CD4_Low_ group) progressed to CD4 counts below 250 cells/μl within 2 years, while the other group (CD4_High_ group) of seven patients maintained CD4 counts above 450 cells/μl. The progression of primary HIV-1 infection can be depicted in six discrete stages, as proposed by Fiebig, et al. [11, 12] Table 1 shows the staging method based on the sequence in emergence of viral marks. All 12 patients had not received antiviral therapy, were in Fiebig stage III–IV and were estimated to have been infected for 1 month at the time of their first positive HIV-1 test. [12, 13] Blood samples were collected at 1, 2, 3, 4, 6, 12 and 24 months after HIV-1 infection. The demographic and clinical characteristics of the 12 men are reported in Table 2.

**Table 1:** Laboratory stages of primary HIV infection based on the emergence of viral markers

**Stage**	**RNA**	**P24 antigen**	**Antibody (ELISA)**	**Western blot**
I	+	–	–	–
II	+	+	–	–
III	+	+	+	–
IV	+	+/–	+	I
V	+	+/–	+	+^b^
VI	+	+/–	+	+

I, indeterminate;

b, without p31 band

**Table 2:** Patient Characteristics.

**Patient**	**Age (year)**	**Fiebig Stage**	**Initial CD4 count (cells/μl)**	**Last CD4 count (cells/μl)**	**VL set point (copies/ml)**	**Days from the initial positive point to CD4<200 cells/μl**
1	22	III	614	181	30,800	714
2	23	III	296	159	24,600	459
3	23	IV	314	188	28,400	196
4	25	IV	327	171	79,600	169
5	26	V	415	117	153,600	153
6	22	III	792	605	662	/
7	23	III	598	714	9,700	/
8	23	III	716	527	7,210	/
9	24	IV	805	827	35,900	/
10	24	IV	603	689	527	/
11	25	V	552	865	1,040	/
12	22	V	678	622	3260	/

VL: viral load

### Ethics statement

The study was approved by the Beijing You’an Hospital Research Ethics Committee, and written informed consent was obtained from each participant.

### Plasma Caspase-1 and Caspase-3 monitoring

Plasma caspase levels were measured using a double antibody sandwich ELISA. Ninety-six well plates were coated with unlabeled polyclonal antibodies to caspase-1 or -3 (Santa Cruz Biotechnology, USA) in 50 μl of antibody solution (20 μg/ml in PBS) and incubated for 2 h at room temperature (RT). After incubation, the wells were washed with PBS-Tween-20 washing buffer, and 100 μl blocking buffer (3% BSA / PBS + 0.02% sodium azide) was added to all of the wells and incubated for 2 h at RT. This step was followed by another washing step and addition of the sample (50 μl), which was incubated at RT for 2 h. The plates were washed, and 50 μl of the polyclonal antibody to Caspase-1 or -3 was added to the respective plates. After a 2-h incubation at RT, a horseradish peroxidase (HRP)-labeled antibody was added to the wells (anti-rabbit HRP for Caspase-1 and anti-goat HRP for Caspase-3) (Sigma, USA). The plates were again incubated for 2 h at RT and washed at the end of incubation. Ortho phenylene diamine (OPD, Sigma, USA) was used as a substrate. The anti-human Caspase-1 used was non-cross reactive with Caspase-1 p10 or Caspase-1 p20. It did not detect Caspase-1 of mouse or rat origin. The anti-human Caspase-3 used was known to react with the carboxy terminal prodomain of caspase-8 (also designated as pro Mch5, MACH alpha 1 or FLICE) of human origin.

### HIV-1 viral load

The viral load (VL) in plasma (copies per milliliter of plasma) was quantified using a nucleic acid sequence-based amplification (NASBA, bioMerieux BV, Boxtel, Netherlands). The assay selectively and directly amplifies HIV-1 RNA in an isothermal, one-step sandwich hybridization procedure using two oligonucleotide primers, three enzymes, nucleoside triphosphates and the appropriate buffers, as previously described. The sensitivity of viral RNA detection by this assay is 50 copies/ml of plasma.

### CD4+ T cell counts

The T lymphocyte counts were determined by three-color flow cytometry using human CD3+, CD4+ and CD8+ cell markers (BD Bioscience, San Diego, CA, USA) in the whole peripheral blood samples from each patient using the FACS lysing solution (Becton Dickinson, San Diego, CA, USA), according to the manufacturer’s instructions. The number of CD4+ T cells per mm^3^ of whole blood was determined.

### Statistical analysis

The Spearman rank test with linear regression was used for the correlation analyses. P values less than 0.05 were considered statistical significant. All statistical operations were performed using SPSS for windows 17.0(SPSS Inc, Chicago, IL).

## Results

### Longitudinal changes in Caspase-1 and Caspase-3 in the CD4_High_ patient group

**Fig 1:**
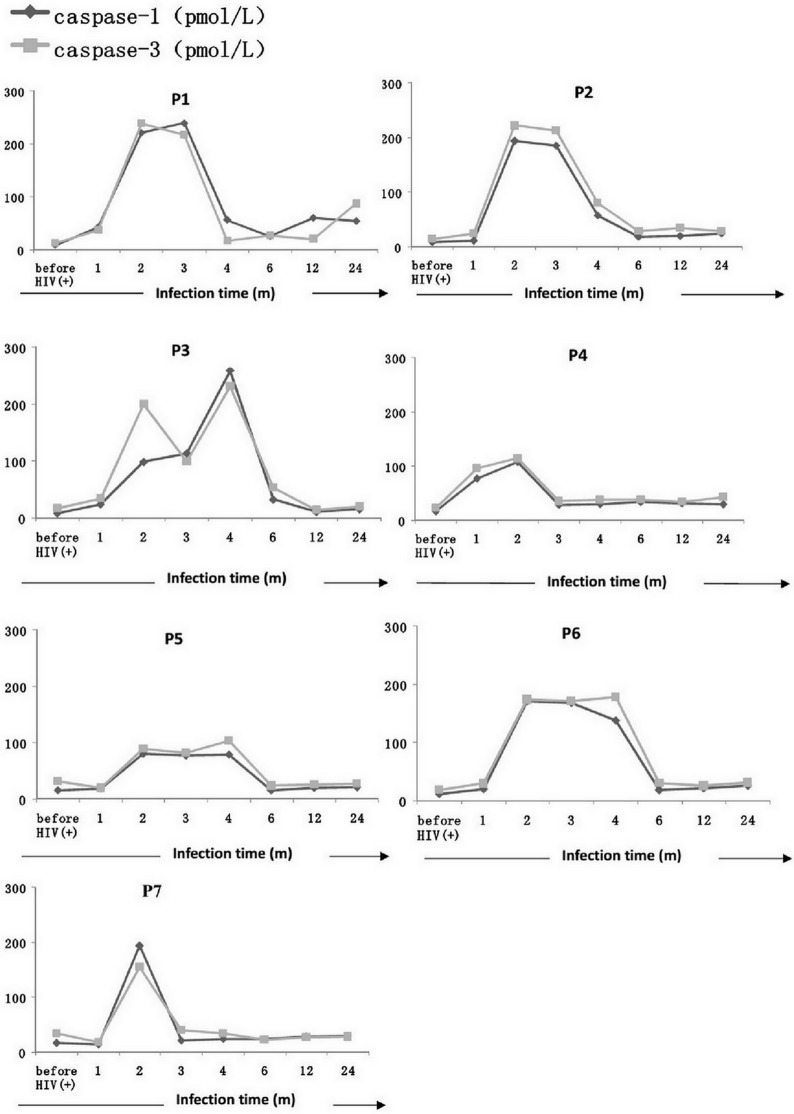
Longitudinal changes in Caspase-1 and Caspase-3 plasma levels in the CD4_High_ patient group. Plasma Caspase-1 and Caspase-3 levels of all the seven patients in the CD4High group (patients 1–7) increased during early HIV-1 infection(six months post-infection) compared with the levels of before HIV-1 infection and then decreased within two years after HIV-1 infection.

In all seven of the men (patients 1–7) in the CD4_High_ group, Caspase-1 and Caspase-3 levels increased during early HIV-1 infection (within 6 months of infection) compared with before HIV-1 infection and then decreased within two years of HIV-1 infection (**Fig. 1**).

### Longitudinal changes in Caspase-1 and Caspase-3 in the CD4_Low_ patient group

**Fig 2:**
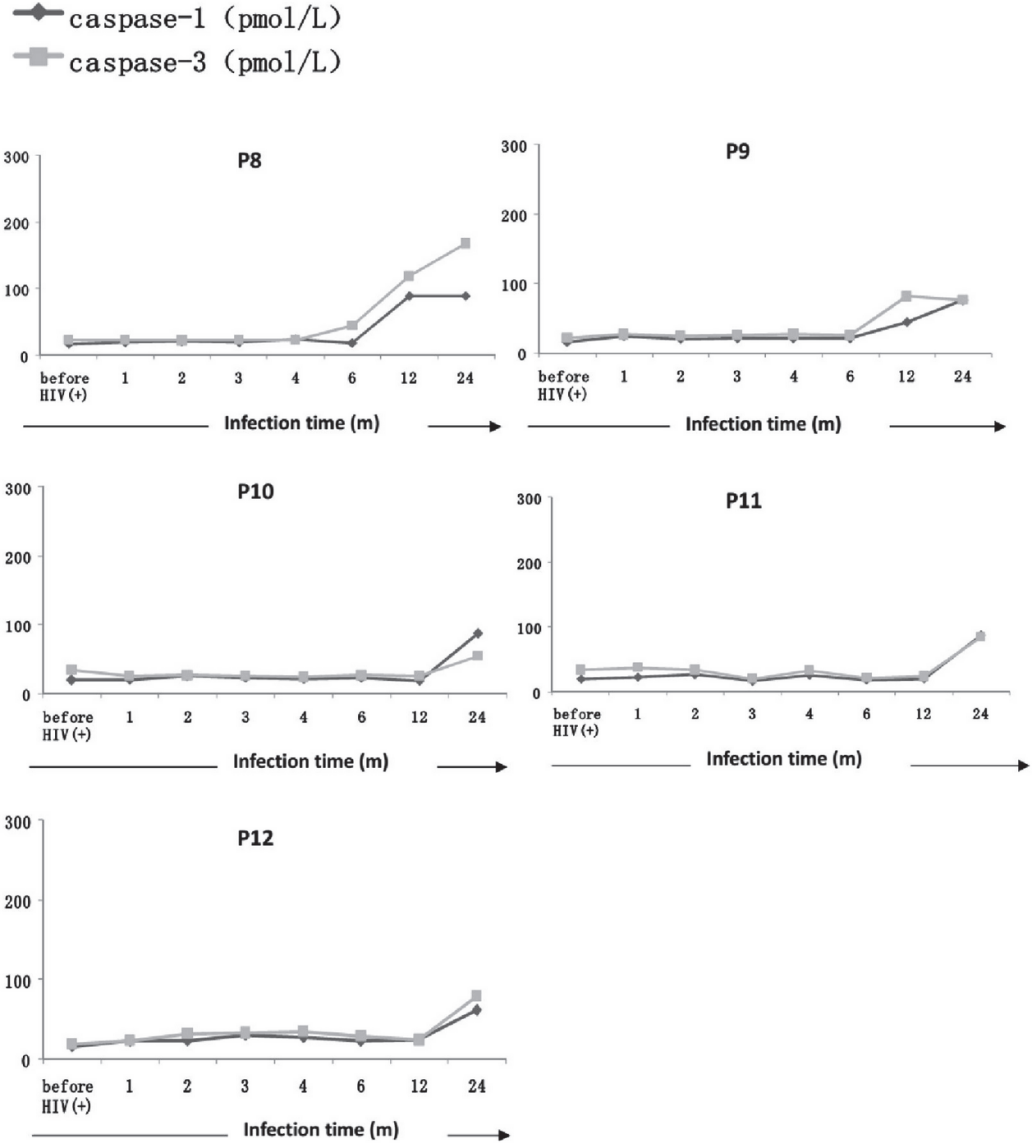
Longitudinal changes in Caspase-1 and Caspase-3 plasma levels in the CD4_Low_ patient group. Plasma Caspase-1 and Caspase-3 levels of all the seven patients in the in the CD4Low group (patients 8–12) did not increase during early HIV-1 infection compared with the levels of before HIV-1 infection (Fig. 2). Caspase-1 and Caspase-3 levels in patient 8 and patient 9 showed an obvious increase 6 months after HIV-1 infection; The levels of Caspase-1 and Caspase-3 in patients 10, 11 and 12showed an obvious increase 12 months after HIV-1 infection.

In all of the five men (patients 8–12) in the CD4_Low_ group, Caspase-1 and Caspase-3 levels did not increase during early HIV-1 infection (within 6 months) compared with before HIV-1 infection (**Fig. 2**). Caspase-1 and Caspase-3 levels in two of the patients (patient 8 and patient 9) showed an obvious increase 6 months after HIV-1 infection (**Fig. 2**). The levels of Caspase-1 and Caspase-3 in three of the patients (patients 10, 11 and 12) showed an obvious increase 12 months after HIV-1 infection (**Fig. 2**).

### Caspase-1 and Caspase-3 plasma levels were positively associated with CD4 counts and negatively associated with VL

**Fig 3:**
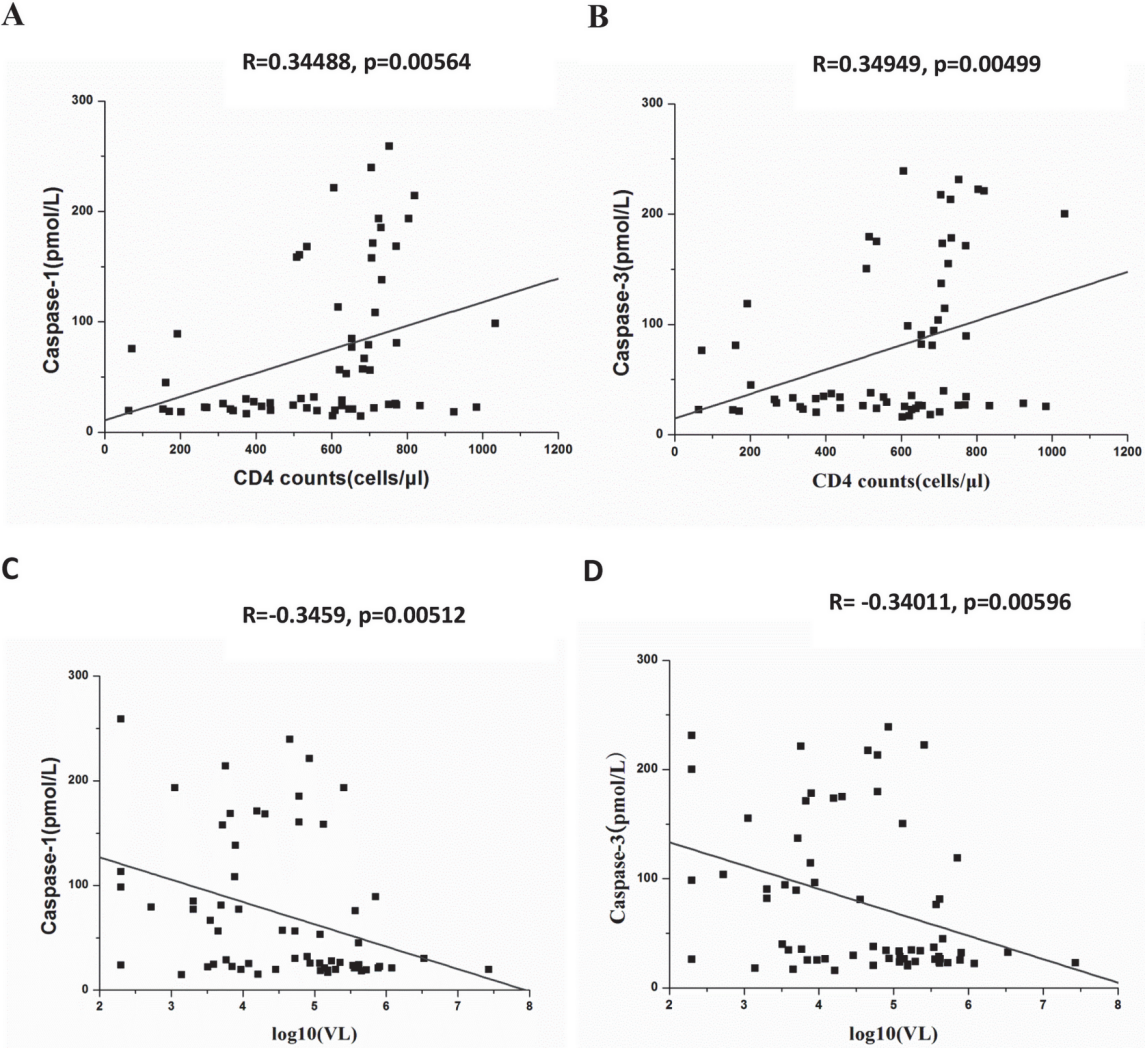
The associations between Caspase-1/3 plasma levels and VL or CD4 counts. Caspase-1 and Caspase-3 plasma levels are positively associated with CD4 counts (R = 0.34488, p = 0.00564 for Caspase-1 and R = 0.34949 p = 0.00499 for Caspase-3; A and B), and negatively associated with VL (R = -0.3459, p = 0.00512 for Caspase-1 and R = -0.34011, p = 0.00596 for Caspase-3; C and D)

We also analyzed the correlations between Caspase-1/3 plasma levels and VL or CD4 counts. Considering the small sample size of each group, the data for both groups and of all time points were pooled together. We found that Caspase-1 and Caspase-3 plasma levels are positively associated with CD4 counts (R = 0.34488, p = 0.00564 for Caspase-1 and R = 0.34949, p = 0.00499 for Caspase-3; Fig. 3A and 3B), and negatively associated with VL (R = -0.3459, p = 0.00512 for Caspase-1 and R = -0.34011, p = 0.00596 for Caspase-3; Fig. 3C and 3D). However, this correlation was weak. The small number of patients is likely partially responsible for the weak correlation.

## Discussion

In this study, we first reported the dynamics of Caspase-1 and Caspase-3 levels in the peripheral blood within 2 years of HIV-1 infection in two distinct patient groups. We found that Caspase-1 and Caspase-3 dynamics were very different between the two groups. Caspase-1 and Caspase-3 levels in the CD4_High_ group first underwent a rapid and robust increase and then decreased after a short time during early HIV-1 infection. It was reported that there is an intense early cytokine storm in acute HIV-1 infection [14]. Bosinger and colleagues reported that there was a rapid robust innate response in SIV-infected sooty mangabeys [15]. Jacquelin and colleagues reported that nonpathogenic SIV infection of African green monkeys induced a strong but rapidly controlled type I **i**nterferon response [16]. We also found that many cytokine levels were higher in the CD4_High_ group than in the CD4_Low_ group during acute HIV-1 infection [10], These cytokines are mainly type I interferon and interferon-induced protein. The increased cytokines in the CD4_High_ group decreased to normal level within a short time (S1 Fig.). These results are consistent with reports from animal studies of SIV infection [15, 16]. Therefore, it is possible that a strong immune response and a large number of cytokines led to increased Caspase-1 and Caspase-3 levels during acute HIV-1 infection in the CD4_High_ group. Caspase-1 and Caspase-3 levels are not noticeably increased during acute HIV-1 infection in the CD4_Low_ group, which may be the result of a weak immune response in the CD4_Low_ group during acute HIV-1 infection. One year after HIV-1 infection, Caspase-1 and Caspase-3 levels were increased in the CD4_Low_ group but not in the CD4_High_ group. It is possible that the translocation of microbial products across a compromised gastrointestinal barrier in the rapid progressors(CD4_Low_ group) was higher after 1 year of HIV-1 infection [17]. The lamina propria CD4+ T cells that are exposed to commensal bacteria were reported to increase the productive infection in the lamina propria CD4+ T cells in vitro by enhancing T cell activation [18]. The activated CD4+ T cells are more susceptible to being infected and are susceptible to cell death. Steele and colleagues have observed increased lamina propria CD4+ T cell depletion in the presence of commensal bacteria in vitro [19]. The microbial translocation that results from a compromised gastrointestinal barrier in late infection may accelerate the progress of the disease by inducing immune cell death.

Viral infections elicit diverse responses including activation of the innate immune system, inflammation and cell death. Both pyroptosis and apoptosis are programmed cell death mechanisms, but they are dependent on different caspases. Caspase-3 is a crucial effector caspase, which cleaves the target molecules that promote apoptosis. Pyroptosis is triggered by Caspase-1 after its activation by various inflammasomes and results in lysis of the affected cell. This mode of cell death is predicted to be inherently inflammatory. Activation of Caspase-1 promotes the maturation and secretion of the inflammatory cytokines interleukin-1β (IL-1β) and IL-18, which results in a worse inflammatory response. It is unclear whether cell death is protective for the host or, on the contrary, favorable for pathogen dissemination in HIV infection. By inducing cell death during infection, the host is effectively eliminating a pathogenic niche and limiting viral replication. However, by inducing cell death, the virus is eliminating host immune cells, and thus weakening the immune response. Our results show that Caspase-1 and Caspase-3 plasma levels are positively associated with CD4 counts and negatively associated with VL. Cell death in early HIV infection may provide protection for the host. Caspase-1 and Caspase-3 levels in the CD4_High_ group increase robustly in acute HIV infection and the infection is soon under control. This may indicate that cell death in early infection promotes viral clearance and delays disease progression. In contrast, the Caspase-1 and Caspase-3 levels in the CD4_Low_ group increased much later. The rapid disease progression of the patients in the CD4_Low_ group is likely associated with the death of immune cells later in the infection.

In conclusion, in this study, we analyzed the dynamics of Caspase-1 and Caspase-3 in the peripheral blood of two distinct patient groups within 2 years of HIV-1 infection. The dynamics of Caspase-1 and Caspase-3 were different between the two groups. The conclusion needs to be further confirmed in a larger population, and the mechanism requires further study. The limitation of the study include that it measured the level of caspase-1 and 3 of in the plasma but not that in CD4 +T cells and Some of the caspases being measured may be inactive. Further research with larger Sample size on the active caspase-1and 3 in CD4+T cells is needed.

## Supporting Information

S1 Fig Cytokines were higher in CD4_High_ group than CD4_Low_ group during acute HIV infection. The levels of IFN-2, IL-1β, IL-2, IL-12, IL-15, FGF-2and VEGF were higher in the CD4_High_ group than in the CD4_Low_ group during Fiebig stages III-IV. After Fiebig stage V, these cytokines decreased to normal levels in the CD4_High_ group but remained elevated in the CD4_Low_ group.
(TIF)
