# Supplementary figures and images for: Longitudinal Changes in Plasma Caspase-1 and Caspase-3 during the First 2 Years of HIV-1 Infection in CD4Low and CD4High Patient Groups
Source: PLoS One. 2015 Mar 25;10(3):e0121011. doi: 10.1371/journal.pone.0121011 (PMC4373860; doi:10.1371/journal.pone.0121011)

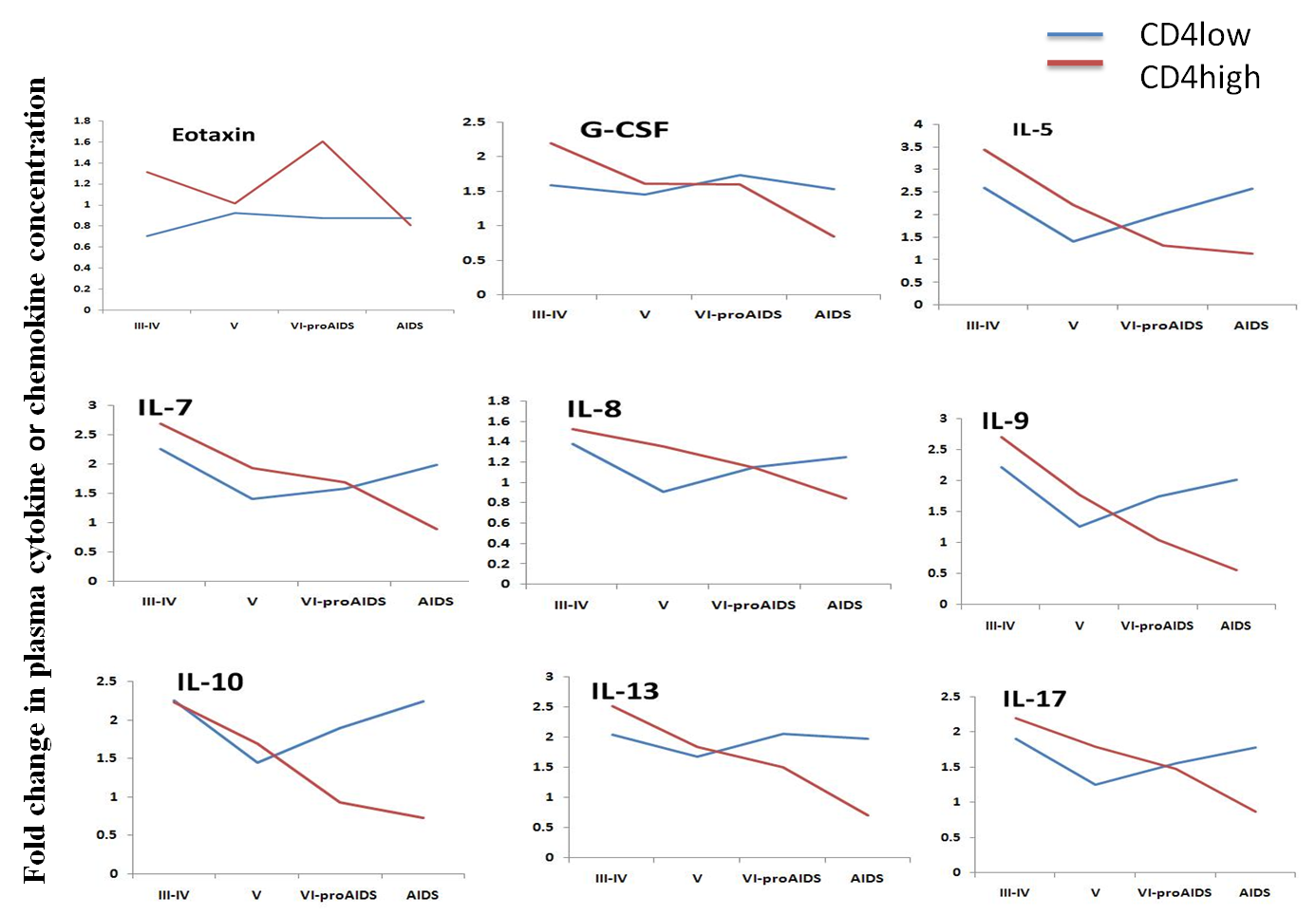

Supplement: S1 Fig — The levels of IFN-2, IL-1β, IL-2, IL-12, IL-15, FGF-2and VEGF were higher in the CD4High group than in the CD4Low group during Fiebig stages III-IV. After Fiebig stage V, these cytokines decreased to normal levels in the CD4High group but remained elevated in the CD4Low group. (TIF) [file pone.0121011.s001.tif]
